# High level expression and biochemical characterization of an alkaline serine protease from *Geobacillus stearothermophilus* to prepare antihypertensive whey protein hydrolysate

**DOI:** 10.1186/s12896-021-00678-7

**Published:** 2021-03-11

**Authors:** Chang Chang, Siyi Gong, Zhiping Liu, Qiaojuan Yan, Zhengqiang Jiang

**Affiliations:** 1 Key Laboratory of Food Bioengineering (China National Light Industry), College of Food Science and Nutritional Engineering, China Agricultural University, No. 17 Qinghua Donglu, Beijing, 100083 China; 2 Beijing Advanced Innovation Center for Food Nutrition and Human Health, College of Engineering, China Agricultural University, Beijing, 100083 China

**Keywords:** Alkaline serine protease, *Geobacillus stearothermophilus*, *Bacillus subtilis*, Whey protein hydrolysate, ACE inhibitory activity

## Abstract

**Background:**

Proteases are important for hydrolysis of proteins to generate peptides with many bioactivities. Thus, the development of novel proteases with high activities is meaningful to discover bioactive peptides. Because natural isolation from animal, plant and microbial sources is impractical to produce large quantities of proteases, gene cloning and expression of target protease are preferred.

**Results:**

In this study, an alkaline serine protease gene (GsProS8) from *Geobacillus stearothermophilus* was successfully cloned and expressed in *Bacillus subtilis*. The recombinant GsProS8 was produced with high protease activity of 3807 U/mL after high cell density fermentation. GsProS8 was then purified through ammonium sulfate precipitation and a two-step chromatographic method to obtain the homogeneous protease. The molecular mass of GsProS8 was estimated to be 27.2 kDa by sodium dodecyl sulphate-polyacrylamide gel electrophoresis (SDS-PAGE) and 28.3 kDa by gel filtration. The optimal activity of GsProS8 was found to be pH 8.5 and 50 °C, respectively. The protease exhibited a broad substrate specificity and different kinetic parameters to casein and whey protein. Furthermore, the hydrolysis of whey protein using GsProS8 resulted in a large amount of peptides with high angiotensin-I-converting enzyme (ACE) inhibitory activity (IC_50_ of 0.129 mg/mL).

**Conclusions:**

GsProS8 could be a potential candidate for industrial applications, especially the preparation of antihypertensive peptides.

**Supplementary Information:**

The online version contains supplementary material available at 10.1186/s12896-021-00678-7.

## Background

Microbial serine proteases (EC 3.4.21.14) are one of most important proteases to be applied in different food fields, such as meat tenderization, cheese ripening, flavor development, baking, and preparation of bioactive peptides [1]. They are composed of serine residue forming a catalytic triad with aspartic acid and histidine in the active site, which can be inactivated by phenylmethylsulfonylfluoride (PMSF), diodopropyl fluorophosphate (DFP) and chymostatin [2]. Alkaline serine proteases have mainly been produced by *Bacillus* species, which have abilities to tolerate pH variance to secrete much amount of proteases (> 20 g/L protein) [1]. Serine proteases from *Bacillus gibsonii*, *Bacillus subtilis* KJ-21 and *Bacillus licheniformis* have been patented to be suitable for use in cleaning fabrics, producing fermented food, and hydrolyzing milk protein to prepare hypoallergenic formula, respectively [3–5].

*Geobacillus stearothermophilus* is extensively distributed in the soil and hot spring, and is a rich source of proteases [6–8]. An alkaline serine protease from *G. stearothermophilus* F1 (presented in composed oil palm branches) was firstly reported by Rahman and coworkers [9], who had further cloned and expressed the gene in *Escherichia coli* [7]. Thereafter, *Pichia pastoris* was used for secretory expression of the protease gene from *G. stearothermophilus* F1, to solve the formation of inclusion bodies and incorrect protein folding induced by *E. coli* expression system, but the expression level was still low (protease activity of 4.13 U/mL) [10]. Meanwhile, another alkaline serine protease from *G. stearothermophilus* strains B-1172 has also been cloned and expressed in *E. coli* to exhibit protease activities of 69 U/mL [8]. As high protease activity and pH stability are important for industrial applications, a proper expression system will be essential to improve the expression level of the target protease genes from *G. stearothermophilus* [1].

*Bacillus subtilis*, a generally recognized as safe (GRAS) bacterium, is widely used as an expression host to secrete foreign proteases directly into culture medium. It is considered as an efficient expression system with several advantages, such as rapid growth rate to result in short fermentation cycles, distinguished ability to secrete significant amounts of proteins into the extracellular medium, easy cultivation and genetic manipulation [1]. A variety of proteases have been successfully expressed in *B. subtilis*, including an alkaline protease (AprE) from *Bacillus licheniformis* 2709 [11, 12], a serine protease (AprB) from *Bacillus* sp. strain B001 [13], a neutral protease (NprT) from *G. stearothermophilus* [14], and alkaline serine proteases from *Bacillus clausii* (aprE) [15, 16]. It has been reported that an alkaline protease from *B. clausii* yielded a protease activity of 1020 U/mL in *B. subtilis* as compared to 347 U/mL in the wild type strain [15]. A thermolabile alkaline protease from *B. licheniformis* 2709 showed a high protease production of 6280 U/mL when it was expressed in *B. subtilis* [11]. However, some of *Bacillus* species directly secreted alkaline serine proteases to present high protease activities without expression in *B. subtilis*. Two proteases (SAPB and SAPRH) were hyper-produced (6500 U/mL and 9000 U/mL) from *Bacillus pumilus* strain CBS and *Bacillus safensis* RH12 under optimized fermentations, as compared with crude protease activities of 310 U/mL and 450 U/mL, respectively [17, 18].

Protein hydrolysis to yield bioactive hydrolysates and peptides is an interesting field with much attentions. Antioxidant, antihypertensive, and antidiabetic potentials are most commonly reported for protein hydrolysates, which may be applied as nutraceuticals and functional food ingredients, potentially contributing to food quality and promoting human health [19]. Particularly, antihypertensive hydrolysates inhibiting angiotensin-I-converting enzyme (ACE), which is very relevant in the regulation of the cardiovascular function and blood pressure, have been abundantly investigated [1]. Recent studies have been focused on the production of antihypertensive hydrolysates using non-commercial proteases, such as alkaline serine proteases from *Bacillus* sp. CL18 [20], *Maclura pomifera* [21], and *Cucurbita ficifolia* [22].

The objective of this study was to clone and express a serine protease gene from *G. stearothermophilus* CAU209 (GsProS8) in *B. subtilis* to improve its activity (as compared with 69 U/mL in *E. coli*) [8]. The recombinant protease was then purified and characterized to evaluate its potential application in the preparation of antihypertensive hydrolysates.

## Results

### Expression and purification of GsProS8

The open reading frame (ORF) of GsProS8 with 1149 bp encoding 383 amino acids was cloned (Fig. S1). The mature protein had a predicted molecular mass of 39.0 kDa. There was no signal peptide in the deduced amino acid sequence based on the analysis by SignalP 4.0. According to the molecular mass determination, a pro-peptide of 107 amino acids was predicted. Thus, the amino acid sequence of GsProS8 consisted of the pro-peptide sequence (1–107) and the mature peptide sequence (108–383). It was found the amino acid sequence of GsProS8 was same as an alkaline serine protease from *G. stearothermophilus* B-1172 (GenBank: EU181368) that was expressed in *E. coli* BL21 [8].

**Fig. 1:**
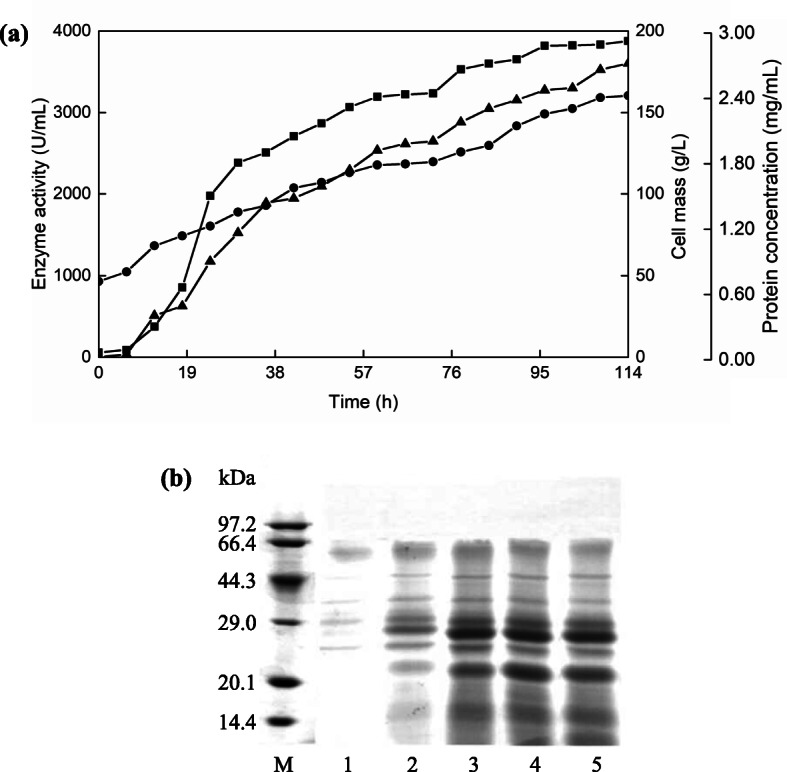
High cell density fermentation of GsProS8 expressed in *B. subtilis*. **a** enzyme activity ( ), protein concentration ( ), and cell mass ( ) of the supernatants. **b** extracellular protein analysis by SDS-PAGE during fermentation. Lane M, the protein marker; lanes 1–5, the supernatants collected at 24 h, 48 h, 72 h, 96 h, and 114 h, respectively. The full-length gel was presented in Fig. S2

Thereafter, GsProS8 was successfully expressed in *B. subtilis* WB600 under the control of P43 promotor [14]. *B. subtilis* WB600 transformant was then cultivated in a 5 L fermentor. The protease activity and protein content showed a continuous increment with time, up to a maximum of 3807 U/mL and 2.08 mg/mL at 114 h, respectively (Fig. 1a). By sodium dodecyl sulphate-polyacrylamide gel electrophoresis (SDS-PAGE), a major protein band around 27 kDa was detected in the fermented medium (Fig. 1b).

**Fig. 2:**
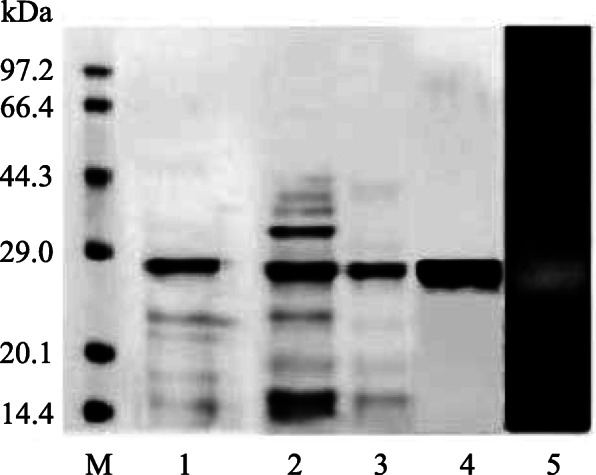
SDS-PAGE analysis of GsProS8 before and after purification. Lane M, the protein marker; lane 1, the crude proteins; lane 2, the proteins purified by ammonium sulphate precipitation; lane 3, the proteins purified by SPFF treatment; lane 4, the proteins purified by QSFF treatment; lane 5, the zymogram of the protease. The full-length gel was presented in Fig. S3 and Fig. S4

**Fig. 3:**
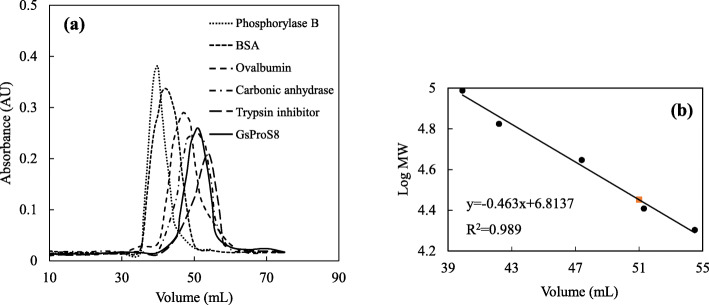
Molecular mass estimation of GsProS8 ( ) by gel filtration chromatography. a: the ultraviolet (UV) chromatogram; b: the calibration curve to calculate molecular mass of GsProS8. Standard proteins (●), including trypsin inhibitor (20.1 kDa), carbonic anhydrase (29.0 kDa), ovalbumin (44.3 kDa), bovine serum albumin (BSA, 66.4 kDa) and phosphorylase B (97.2 kDa)

After the fermentation, GsProS8 was purified 2.1-fold to homogeneity with a yield of 23.6%. The specific activity of the protease was increased from 1679.1 U/mg to 3559.7 U/mg (Table 1). The homogeneity of purified GsProS8 was confirmed via a single smeared band at 27.2 kDa by SDS-PAGE (Fig. 2), while the native molecular mass was determined to be 28.3 kDa by gel filtration (Fig. 3). This indicated that GsProS8 is a monomer. Because the conversion process of the primary gene product into the mature enzyme can be mediated by active subtilisin to cleave off the pro-peptide sequence in the extracellular medium during the secretion [11], the molecular mass of purified GsProS8 was lower than the predicted value of 39.0 kDa. Moreover, the purified GsProS8 was evaluated and a clear band of proteolytic activity was observed in the zymogram (Fig. 2).

**Table 1:** Purification summary of GsProS8

Purification step	Total activity (U)^a^	Total protein (mg)^b^	Specific activity (U/mg)	Purification factor	Yield (%)
Crude protease	116,250 ± 2089.3	68.5 ± 4.1	1697.1 ± 30.2	1.0	100.0
Ammonium sulfate precipitation	86,460 ± 1965.2	41.6 ± 1.9	2078.4 ± 47.5	1.2	74.4
SPFF	50,806 ± 190.5	22.4 ± 5.4	2268.1 ± 9.1	1.3	43.7
QSFF	27,409 ± 373.9	7.7 ± 1.2	3559.7 ± 48.4	2.1	23.6

^a^Enzymatic reaction was carried out using casein as a substrate at 50 °C in 0.05 mol/L MOPS buffer (pH 8.5)

^b^Total protein was measured using BSA as standard by the Lowry method

### Biochemical properties and kinetic parameters of GsProS8

**Fig. 4:**
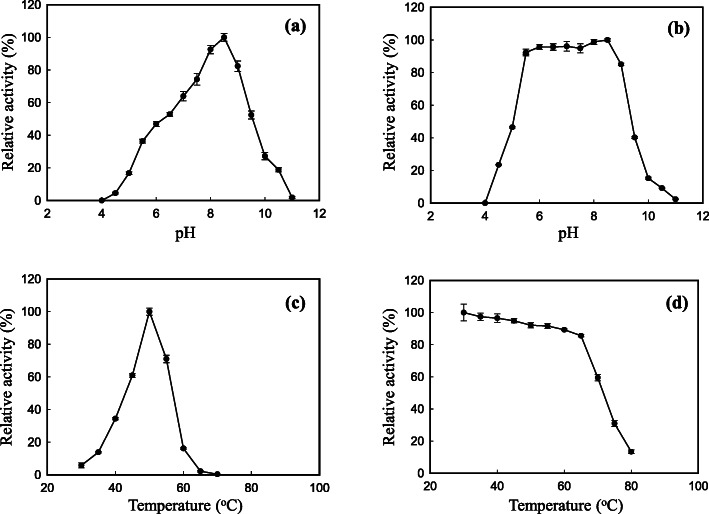
Optimal pH (**a**), pH stability (**b**), optimal temperature (**c**), and thermostability (**d**) of GsProS8. The influence of pH on GsProS8 activity was measured in 0.05 mol/L of different buffers at 40 °C. For the optimal pH, the relative activity (%) was determined against the specific activity of 3916 U/mg. To determine pH stability against the optimal activity (2631 U/mg, defined as 100% relative activity), GsProS8 was incubated in various buffers (0.05 mol/L) at 40 °C for 30 min. For optimal temperature, GsProS8 activity was tested at different temperatures in MOPS buffer (0.05 mol/L, at pH 8.5) in contrast to the specific activity of 3916 U/mg. The thermostability of GsProS8 was assayed against the optimal activity (3915 U/mg, defined as 100% relative activity) after incubation at different temperatures for 30 min

The highest activity of GsProS8 was observed at pH 8.5 (Fig. 4a). The protease was stable at pH 5.5–8.5 to retain more than 90% of the activity (Fig. 4b). The optimal temperature of GsProS8 was found to be 50 °C (Fig. 4c). It was stable up to 65 °C, retaining more than 80% of the activity (Fig. 4d). Protease inhibitors were subjected to identify groups at the active site of GsProS8. Pepstatin A (aspartic protease inhibitor) and iodoacetamide (cysteine protease inhibitor) slightly affected the protease activity, indicating that aspartate residue and -SH group did not work in the protease activity of GsProS8. The presence of ethylenediaminetetraacetic acid (EDTA, metalloenzyme inhibitor) at 1 mM and 4 mM inhibited 30.5 and 40.5% of the protease activity, respectively, revealing that some ions may be important for the stability and activity of GsProS8. The strong inhibition of 95.4 and 99.7% at 1 mM and 4 mM of PMSF (serine protease inhibitor) suggested that GsProS8 belongs to the serine protease class (Table 2).

**Table 2:** Effect of selective protease inhibitors on the protease activity of GsProS8. Different letters (a-f) on the same column indicated significant (*p* < 0.05) differences among samples*

Inhibitor	Concentration (mM)	Specific activity (U/mg)	Relative activity (%)
Pepstatin A	0.01	3201.0 ± 49.7^a^	89.9
	0.02	2993.5 ± 52.0^b^	84.1
PMSF	1	162.3 ± 20.3^e^	4.6
	4	10.3 ± 2.3^f^	0.3
Iodoacetamide	1	3277.0 ± 15.8^a^	92.1
	4	2901.0 ± 36.2^b^	81.5
EDTA	1	2475.7 ± 29.4^c^	69.5
	4	2118.2 ± 6.8^d^	59.5

*Enzymatic reaction was carried out using casein as a substrate at 50 °C in 0.05 mol/L MOPS buffer (pH 8.5). Specific activity was shown as mean ± SD (*n* = 3). Relative activity was expressed as a percentage of the activity in the absence of inhibitors (the control)

The effects of various metal ions on the protease activity were assessed (Table 3). The protease activity of GsProS8 was not affected by Mg^2+^, but slightly impacted by Ca^2+^ (93.7%) and Na^+^ (91.4%). The moderate reduction in the protease activity of GsProS8 was observed in the presence of Mn^2+^ (78.9%), Zn^2+^ (78.1%), Co^2+^ (76.6%), Ni^2+^ (66.1%), and Cu^2+^ (40.7%). In addition, GsProS8 exhibited the highest protease activity towards casein (100%), followed by whey protein (92.1%) and skim milk powder (85.5%). The protease showed a broad substrate specificity towards hemoglobin (60.7%), soybean protein isolate (56.7%), bovine serum albumin (BSA, 45.7%), protamine (37.5%), myoglobin (36.9%), and azocasein (31.2%). However, GsProS8 displayed low activity towards gelatin (10.5%) (Table 4). The kinetic parameters of the purified GsProS8 were determined using casein and whey protein as substrates. The values of *K*_m_ and *V*_max_ were 7.37 mg/mL and 13.35 mg/mL, 231.45 μmol/mg·min and 122.96 μmol/mg·min for casein and whey protein, respectively (Table 5).

**Table 3:** Effect of metal ions on the protease activity of GsProS8. Different letters (a-g) on the same column indicated significant (*p* < 0.05) differences among samples*

Metal ion	Specific activity (U/mg)	Relative activity (%)
None	3559.7 ± 48.4^a^	100.0
Mg^2+^	3593.4 ± 2.3^a^	100.9
Ca^2+^	3334.5 ± 15.8^b^	93.7
Na^+^	3254.4 ± 18.1^c^	91.4
Ni^2+^	2354.5 ± 9.0^f^	66.1
Mn^2+^	2808.6 ± 24.9^d^	78.9
Co^2+^	2726.4 ± 5.0^e^	76.6
Zn^2+^	2779.8 ± 6.8^de^	78.1
Cu^2+^	1450.5 ± 9.0^g^	40.7

*Enzymatic reaction was carried out using casein as a substrate at 50 °C in 0.05 mol/L MOPS buffer (pH 8.5). Specific activity was shown as mean ± SD (*n* = 3). Relative activity was expressed as a percentage of the activity in the absence of metal ions (the control)

**Table 4:** Substrate specificity of GsProS8. Different letters (a-h) on the same column indicated significant (*p* < 0.05) differences among samples*

Substrate	Specific activity (U/mg)	Relative activity (%)
Casein	3560.5 ± 15.8^a^	100
Whey protein	3277.0 ± 19.8^b^	92.1
Skim milk powder	3044.8 ± 13.6^c^	85.5
Hemoglobin	2161.4 ± 54.2^d^	60.7
Soybean protein isolate	2025.8 ± 45.2^d^	56.9
Bovine serum albumin	1627.2 ± 22.6^e^	45.7
Protamine	1333.4 ± 6.8^f^	37.5
Myoglobin	1312.9 ± 33.9^f^	36.9
Azocasein	1109.5 ± 0.1^g^	31.2
Gelatin	374.1 ± 1.6^h^	10.5

*Enzymatic reaction was carried out using different substrates at 50 °C in 0.05 mol/L MOPS buffer (pH 8.5). Specific activity was shown as mean ± SD (*n* = 3). Relative activity was expressed as a percentage of the activity when casein as a substrate (the control)

**Table 5:** Kinetic parameters of GsProS8 for casein and whey protein. Different letters (a-b) on the same column indicated significant (*p* < 0.05) differences among samples*

Substrate	***V***_**max**_ (μmol/mg·min)	***K***_**m**_ (mg/mL)	***k***_**cat**_ (s^**−1**^)	***k***_**cat**_/***K***_**m**_ (mL/mg·s)
Casein	231.50 ± 3.07^a^	7.37 ± 0.24^b^	0.105	0.014
Whey protein	122.96 ± 4.98^b^	13.35 ± 1.17^a^	0.056	0.004

*The measurement of kinetic parameters was carried out using casein and whey protein as substrates at 50 °C in 0.05 mol/L MOPS buffer (pH 8.5). *K*_m_ and *V*_max_ were shown as mean ± SD (*n* = 3)

### Preparation of antihypertensive whey protein hydrolysates by GsProS8

The optimization of whey protein hydrolyzation by GsProS8 based on the orthogonal experimental design is shown in Table 6. The correlation coefficient value (R) demonstrated that whey protein concentration was the most affective factor (R = 19.1) on the peptide contents of whey protein hydrolysates, followed by temperature, pH and time. In addition, higher K value in each column showed the stronger impacts of the level (1, 2, or 3) on the peptide contents. Therefore, evaluated by K values, the optimized hydrolysis conditions to prepare whey protein hydrolysate by GsProS8 should be performed using 11% (w/v) of whey protein at pH 8.0 and 60 °C for 6 h.

**Table 6:** Orthogonal experimental results and the optimization of whey protein hydrolysates by GsPros8

Trial	Factor				Peptide content (%)
	pH	Temperature (°C)	Time (h)	Whey protein concentration (%)	
1	1	1	1	1	15.6
2	2	1	2	2	17.0
3	3	1	3	3	21.7
4	1	2	2	3	33.4
5	2	2	3	1	29.2
6	3	2	1	2	34.9
7	1	3	3	2	31.4
8	2	3	1	3	51.5
9	3	3	2	1	14.5
K_1_^a^	23.5	14.8	21.6	16.4	
K_2_^a^	32.6	32.5	30.7	27.8	
K_3_^a^	23.7	32.5	27.4	35.5	
R^b^	9.1	17.7	9.0	19.1	
The impact of factors	Whey protein concentration > Temperature > pH > Time				
Optimized hydrolysis condition	pH 8.0, 60 °C, 6 h, 11% of whey protein concentration				

^a^K_1_, K_2_, and K_3_ indicated the sum of peptide contents corresponding to level 1, level 2, and level 3, respectively

^b^R was the correlation coefficient value, R = Max K_i_ – Min K_i_ (i = 1, 2, or 3)

**Fig. 5:**
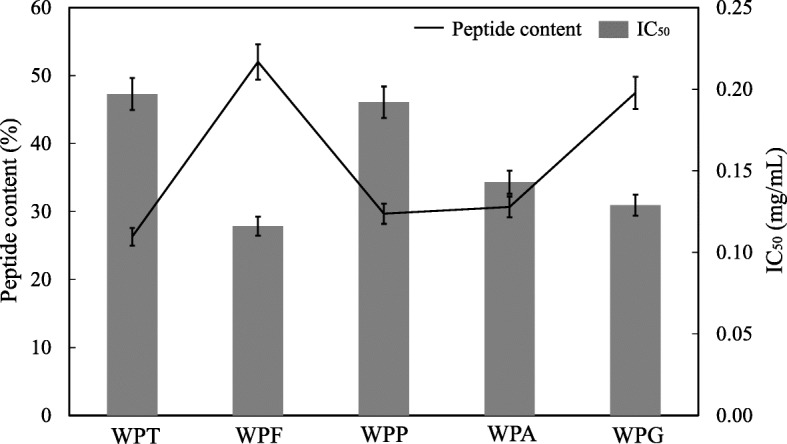
Peptide contents and IC_50_ values of whey protein hydrolysates prepared by GsProS8 versus commercial proteases. WPT, whey protein hydrolysate prepared by trypsin at pH 8.0 and 37 °C; WPF, whey protein hydrolysate prepared by Flavourzyme at pH 7.0 and 53 °C; WPP, whey protein hydrolysate prepared by Protamex at pH 7.5 and 40 °C; WPA, whey protein hydrolysate prepared by Alcalase at pH 8.0 and 60 °C; WPG, whey protein hydrolysate prepared by GsProS8 at pH 8.0 and 60 °C

The performance of GsProS8 was compared with commercial proteases in the preparation of antihypertensive whey protein hydrolysates (Fig. 5). The whey protein hydrolysate prepared by Flavourzyme contained the highest (*p* < 0.05) peptide content (52.02%) with the strongest ACE inhibitory activity (IC_50_ of 0.116 mg/mL), followed by the hydrolysates generated by GsProS8 (IC_50_ of 0.129 mg/mL) and Alcalase (IC_50_ of 0.143 mg/mL). Nevertheless, both of trypsin and Protamex were less (*p* < 0.05) suitable to prepare antihypertensive whey protein hydrolysates, because of the higher IC_50_ values (0.197 mg/mL and 0.192 mg/mL).

## Discussion

*G. stearothermophilus* can produce extracellular neutral and alkaline proteases [8, 14]. Many researches have explored alkaline serine proteases from several strains of *G. stearothermophilus*, such as strain F1 [7], strain B-1172 [8], and strain AP-4 [6]. In the present study, an alkaline serine protease gene (GsProS8) from *G. stearothermophilus* CAU209 was cloned and expressed in *B. subtilis* WB600. Although GsProS8 shared the same amino acid sequence with the reported protease from *G. stearothermophilus* B-1172 [8], the protease activity of GsProS8 (3807 U/mL) after high cell density fermentation (Fig. 1a) was much higher than the proteases from the strain AP-4 (250 U/mL) [6], the strain F1 (1500 U/mL) and the strain B-1172 (69 U/mL) that were expressed in *E. coli* [7, 8]. This may be attributed to the detrimental effects on the *E. coli* by the intracellular accumulation of the alkaline proteases [11]. On the contrary, *B. subtilis* is a more attractive expression host to steadily secrete extracellular proteins directly into the culture medium [23]. Moreover, the protease activity of GsProS8 was also higher than the other alkaline serine proteases from *Aspergillus sojae* (400 U/mL) [24], *B. subtilis* RD7 (607 U/mL) [2], and *B. clausii* (1020 U/mL) [25].

After purification, the specific activity of GsProS8 increased from 1697.1 U/mg to 3559.7 U/mg (Table 1), which is much higher than the previously reported proteases from *G. stearothermophilus* strain B-1172 (97.5 U/mg) [8] and strain F1 (1790 U/mg) [7]. In general, the molecular masses of alkaline serine proteases from bacteria have been reported to be 30–45 kDa. Molecular masses of alkaline serine proteases from *A. sojae*, *B. subtilis* RD7, and *Bacillus lehensis* JO-26 were reported to be 35.2 kDa [24], 43 kDa [2], and 34.6 kDa [26], respectively. In this study, the purified GsProS8 had a relative molecular mass of 27.2 kDa on SDS-PAGE and 28.3 kDa in gel filtration (Fig. 3), which presented a good dispersibility and permeability [27]. However, the predicted molecular mass of GsProS8 was same as the protease (100% identity of amino acid sequences) expressed in *E. coli* (39 kDa) [8]. The conversion of the primary gene product into the mature protease is most likely autocatalytic, and the pro-peptide sequence might be cleaved by a self-processing mechanism. Thus, the molecular mass of purified protease did not correspond with the prediction by gene sequence [11]. Tang and co-workers [11] cloned and expressed an alkaline protease gene from *B. licheniformis* 2709 in *B. subtilis* WB600. The truncation of pro-peptide was demonstrated by the molecular mass of 30.5 kDa in contrast to the predicted value of 55 kDa [28]. This truncation has also been reported in the keratinolytic protease from *Thermoactinomyces* sp. YT06, alkaline protease from *Vibrio* sp. DA1–1, and serine protease from *Dichelobacter nodosus*, in which the recombinant proteases were 28–37 kDa, instead of the expected 40.3–50.6 kDa [29–31]. The keratinase and alkaline protease genes from *Thermoactinomyces* sp. YT06 and *Vibrio* sp. DA1–1 were cloned and expressed in *E. coli* BL21, and the pro-sequences were also autoproteolytically cleaved in the periplasm [29, 30]. Therefore, the truncation was not specific to over-expression in *B. subtilis* WB600.

Species of *Bacillus* are the most representative microbial strains used in the production of alkaline serine proteases with many applications in food industry, such as hydrolyzing proteins to obtain bioactive ingredients [32] and modifying wheat gluten to improve the texture of baking products [33]. Notably, the majority alkaline serine proteases exhibit optimal pH in the range of 7 to 11 [27]. The optimal pH 8.5 of GsProS8 is committed to this range, but is slightly lower than the proteases (pH 9.0–10.6) from other strains of *G. stearothermophilus* [6], *Bacillus* sp. strain B001 [13], *B. subtilis* RD7 [2], and *A. sojae* [24]. Meanwhile, the pH stability of GsProS8 was comparable with the protease from *G. stearothermophilus* B-1172, in which a high residual activity (> 87%) was retained after incubation of the proteases at pH 5.0–9.0 [8]. However, the residual activities of the proteases from *G. stearothermophilus* F1 and AP-4 kept 95% of residual activities within pH 8.0–10.0 [6, 7].

Furthermore, GsProS8 had a maximal activity at 50 °C (Fig. 4c), which is lower than those bacterial proteases from *G. stearothermophilus* F1 expressed in *E. coli* (80 °C) and *Bacillus* sp. strain B001 (60 °C) [7, 13]. In theory, the expression and biochemical properties of proteases are influenced by many factors, such as the strain of expression host, culture temperature, the inducing temperature and time [34]. As the protein expressed in *E. coli*, the recombinant protease was secreted within cytoplasm as a soluble and active form to maintain the original living temperature of *G. stearothermophilus*. While expression in *B. subtilis*, the recombinant protease was extracellularly secreted into culture medium at 37 °C. The optimal temperature of GsProS8 expressed in *B. subtilis* was deduced. This was also proved by Zhang and co-workers [14], in which the protease (NprT) from *G. stearothermophilus* expressed in *B. subtilis* DB104 was different from the growth temperature of *G. stearothermophilus* (Table 7). In addition, the lower molecular mass of GsProS8 (27.2 kDa) was contributed to the lower optimal temperature (50 °C) as compared with the protease gene expressed in *E. coli* (39 kDa) [8]. A similar finding was reported by Liu and co-workers [35], who compared keratinase gene expression in *E. coli*, *B. subtilis*, and *P. pastoris*. However, GsProS8 was comparable and displayed an even higher optimal temperature than the proteases from *B. lehensis* JO-26 (50 °C) [26], *A. sojae* expressed in *P. pastoris* (40 °C) [24], and *B. subtilis* RD7 expressed in *E. coli* (40 °C) [2]. Therefore, GsProS8 can be handled for industrial applications under an alkaline condition with a relative high temperature to increase reaction rate.

**Table 7:** Alkaline serine proteases from *G. stearothermophilus* and being used to prepare ACE inhibitory hydrolysates

Origin of alkaline serine protease	Expression host	Molecular mass (kDa)	Protease activity (U/mL)^**a**^	Optimal pH and temperature	Hydrolyzed protein^**b**^	IC_**50**_ (mg/mL)	Reference
*G. stearothermophilus* CAU209	*B. subtilis* WB600	27.2	3807	pH 8.5, 50 °C	Whey protein	0.129	This study
*G. stearothermophilus* AP-4	–	–	250	pH 9.0, 55 °C	–	–	6
*G. stearothermophilus* F1	*E.coli* XL1-Blue	27	1500	pH 9.0, 80 °C	–	–	7
*G. stearothermophilus* B-1172	*E. coli* BL21	39	64	pH 9.0, 90 °C	–	–	8
*G. stearothermophilus*	*B. subtilis* DB104	35	7020	pH 7.5, 65 °C	–	–	14
*Anoxybacillus kamchatkensis* M1V	*E. coli* BL21	28	4600	pH 11.0, 70 °C	Shrimp protein	0.010	44, 45
*Aeribacillus pallidus* VP3	–	29	3000	pH 10.0, 60 °C	Shrimp protein	0.014	45, 46
*Yarrowia lipolytica*	–	35	–	pH 9.0, 45 °C	Egg white protein	1.229	47, 48
*Sardina pilchardus*	–	25	500	pH 8.0, 60 °C	Sardinelle protein	1.200	49, 50
*A. clavatus* ES1	*E. coli* BL21	32	260	pH 8.5, 50 °C	Sardinelle protein	7.400	38, 50
*B. licheniformis* NH1	–	27	1600	pH 10.0, 70 °C	Sardinelle protein	2.100	50, 51

^a^Protease activity was measured using casein as a substrate at the optimal conditions for each protease

^b^The protein was hydrolyzed under the optimal hydrolysis condition of each protease

- Not found

The protease activity of GsProS8 relatively remained constant and was not declined too much in the presence of metal ions, except Cu^2+^ (Table 3). Thus, the protease described in this study is not a metalloenzyme. This is similar with the subtilisins from *B. subtilis* RD7 [2] and *B. lehensis* JO-26 [26], but better than the one from *Bacillus* spp. B001 [13], whose activities were almost completely reduced by Cu^2+^, Zn^2+^, Ni^2+^, and Co^2+^. The protease activity of GsProS8 was affected in the presence of heavy metal Cu^2+^, because it might chelate with the protease causing precipitation and deactivation [36]. In addition, the reduction of protease activity in the presence of Ni^2+^, Mn^2+^, Co^2+^, and Zn^2+^ demonstrated that GsProS8 might contain a number of metal ions, so, the displacement or substitution of them compromise the catalysis [2, 8].

GsProS8 was highly specific towards casein and whey protein (Table 4). This may be resulted from the maximization of the binding energy of casein and whey protein for the protease that decided the substrate affinity [8]. The result is in accordance with the alkaline serine proteases from *G. stearothermophilus* B-1172 and *A. sojae* showing the highest activities towards casein [8, 24]. In the meantime, GsProS8 had significant hydrolysis preferences to skim milk powder, hemoglobin, and soybean protein isolate, and relative activities towards BSA, protamine, myoglobin, and azocasein (Table 4). So, it displayed broader substrate specificity than the proteases from *B. subtilis* RD7 [2], *A. sojae* [24], and *G. stearothermophilus* B-1172 [8]. However, azocasein was found to be the most preferred substrate for the protease from *B. subtilis* RD7 [2].

As summarized in literature, the values of *K*_m_ and *V*_max_ of proteases presented as 0.08–5.10 mg/mL and 25–7692 U/mg, respectively [37–40]. GsProS8 exhibited higher affinity than the other alkaline serine proteases (Table 5), to indicate potential application in commercial products. Additionally, because caseins carried open structures to be susceptible for proteolysis than whey protein with a globular nature [41], the kinetic parameters of GsProS8 acting on whey protein were significantly lower than those on casein.

Proteases have been found to display many applications in the food industry, in which the preparation of protein hydrolysates with remarkable biological activities (e.g., antihypertensive, immunostimulating, antimicrobial, and antioxidant activities) have attracted much attention [1]. Due to excellent nutritional and functional properties, whey protein hydrolysates produced by protease hydrolysis, microbial fermentation, and heat treatment have been widely used in food industry [42]. ACE inhibitory activity of whey protein hydrolysates has been discovered using various proteases (e.g., trypsin, Alcalase, bromelain, papain, and pepsin) [43]. In this study, whey protein was hydrolyzed by GsProS8 and commercial proteases (e.g., trypsin, Flavourzyme, Protamex, and Alcalase), GsProS8 hydrolysate contained higher peptide content and ACE inhibitory activity (Fig. 5). In particular, the whey protein hydrolysate prepared by GsProS8 (IC_50_ of 0.129 mg/mL) exhibited better antihypertensive activity than the hydrolysates produced by other alkaline serine proteases from *Maclura pomifera* (IC_50_ of 0.53 mg/mL) [21] and *Cucurbita ficifolia* (IC_50_ of 0.65 mg/mL) [22]. In addition, GsProS8 was compared with other alkaline serine proteases for the preparation of ACE inhibitory hydrolysates (Table 7). GsProS8 has comparable biochemical properties as compared with alkaline serine proteases from *Anoxybacillus kamchatkensis* strain M1V and *Aeribacillus pallidus* strain VP3. Moreover, GsProS8 hydrolyzed whey protein to yield a lower IC_50_ value (0.129 mg/mL) than the hydrolysates prepared by the proteases from *Yarrowia lipolytica, S. pilchardus*, *A. clavatus* ES1 and *B. licheniformis* NH1 (1.200–7.400 mg/mL) [38, 44–51]. This revealed that GsProS8 has potential to be used for preparation of antihypertensive hydrolysates and peptides.

## Conclusions

In summary, an alkaline serine protease gene from *G. stearothermophilus* CAU209 has been expressed in *B. subtilis* WB600, to exhibit high protease activity (3807 U/mL) after high cell density fermentation. GsProS8 was purified as a monomeric protein with a molecular mass of 27.2 kDa. It showed maximal protease activity at pH 8.5 and 50 °C with a broad substrate specificity. Moreover, GsProS8 displayed a good ability to hydrolyze whey protein to prepare antihypertensive hydrolysates. The properties of GsProS8 provided important insights for its applications into food industry.

## Methods

### Bacterial strain, plasmids and media

*G. stearothermophilus* CAU209 was deposited in Key Laboratory of Food Bioengineering (China National Light Industry) in Beijing, China. *B. subtilis* WB600 (*Δbpr, Δepr, Δmpr, ΔnprB, Δvpr, ΔwprA*), a six extracellular protease deficient strain gifted from Guangxi University, Nanning, China, was used as the expression host. *E. coli* DH5α [*F*^*−*^ *supE44 Ф80 δlacZ ΔM15 Δ (lacZYA-argF) U169 endA1 recA1 hsdR17 (r*_*K*_^*−*^*, m*_*K*_^*+*^*) deoR thi-1 λ- gyrA96 relA1*] (Biomed, Beijing, China) was used as the host strain for DNA manipulation, and was cultured in Luria-Bertani (LB) medium composed of 10 g/L peptone, 5 g/L yeast extract, and 5 g/L NaCl. Plasmid pWB980 (TaKaRa Corporation, Dalian, China) was used as the expression vector. Whey protein (80% of protein content on dry basis) was obtained from Ausnutria Dairy Corporation (Changsha, China). All other chemicals and reagents were of analytical grades and commercially available from Sigma-Aldrich (St. Louis, MO, USA).

### Cloning of the alkaline serine protease gene (GsProS8)

Genomic DNA from *G. stearothermophilus* CAU209 (GenBank: MW084977) was extracted as described previously [52]. According to the genome sequence of *G. stearothermophilus* CAU209, a pair of oligonucleotide primers, named GsProS8-F and GsProS8-R (Table S1), were designed to amplify the coding region of the alkaline serine protease. Polymerase chain reaction (PCR) was performed at 94 °C for 5 min, 30 cycles with denaturing at 94 °C for 30 s, annealing at 56 °C for 30 s and extension at 72 °C for 1.5 min, and the last cycle was at 72 °C for 10 min. The PCR product was ligated with pMD-19 T and transformed into *E. coli* DH5α for sequencing. Sequence analysis was performed using DNAMAN 6.0. The ORF was found using ORF Finder (http://www.ncbi.nlm.nih.gov/gorf/orfig.cgi/). The signal peptide was predicted by SignalP 4.0 (http://www.cbs.dtu.dk/services/SignalP/).

### Plasmid construction for expression of GsProS8

To express the protease gene in *B. subtilis* WB600, two pairs of primers (GsProS8-IF and GsProS8-IR, pWB980-VF and pWB980-VR, Table S1) were designed to amplify the ORF region of the GsProS8 gene on the chromosome DNA and the vector, respectively. The overlapping PCR was carried out using 0.0002 mol/L each of the four dNTPs and 0.04 U Phusion DNA polymerase. P43 promoter was located at the upstream of *SacB* signal peptide in the plasmid pWB980. PCR conditions were as follows: 94 °C for 5 min, 30 cycles of 94 °C for 30 s, 56 °C for 30 s, 72 °C for 1.5 min (the last cycle at 72 °C for 10 min). The purified PCR product was digested with *EcoRI* and *SmaI* restriction endonucleases, and then inserted into the plasmid pWB980. The recombinant plasmid pWB980-GsProS8 was transformed into the *B. subtilis* WB600 by electroporation. The transformants were cultured on LB plates and incubated at 37 °C until colonies appeared. The colonies were then suspended in the LB medium for further growth in a rotary shaker at 37 °C for 18–24 h. The cultures were centrifuged at 10,000×*g* for 10 min to collect the supernatant. The protease activity as well as the expression in the supernatant were analyzed by the Folin-Ciocalteu reagent and SDS-PAGE, respectively.

### High cell density fermentation

To scale up GsProS8 production, high cell density fermentation was carried out in a 5.0 L fermentor (Guoqiang, Shanghai, China) with a 1.5 L working volume at 37 °C. 1% (v/v) inoculum was initially incubated at 37 °C for 12 h. Subsequently, the fermentor was inoculated with 750 mL of the inoculum and cultivated at 37 °C for 114 h. The fermentation medium was composed of glucose (10 g/L), yeast extract powder (10 g/L), peptone (20 g/L), corn steep powder (5 g/L), sodium chloride (10 g/L), magnesium sulfate (0.3 g/L), sodium phosphate (6 g/L), and dipotassium phosphate (3 g/L). The cultivation was maintained at pH 7.0–7.2 with the aid of ammonium hydroxide and phosphoric acid. The dissolved oxygen level was maintained at 30% air-saturation. Samples were withdrawn at 6 h interval to analyze enzyme activity, cell mass, and protein concentration, and SDS-PAGE was also performed.

### Purification of GsProS8

The fermented culture was purified through ammonium sulfate precipitation and a two-step chromatographic method. In short, the crude enzyme was initially precipitated by ammonium sulphate (40–70% w/v saturation) and dialyzed against 0.02 mol/L Tris-HCl at pH 8.0 (buffer A) for overnight. Thereafter, the dialyzed sample was subjected to a HiTrap SP Fast Flow (SPFF) column (GE Healthcare, Wuxi, China), which was pre-equilibrated with buffer A. After washing with buffer A until the absorbance (at 280, OD_280_) < 0.05, the bounded proteins were then eluted with a linear gradient (0–0.25 mol/L) of NaCl at a flow rate of 1 mL/min. The fraction with high protease activity was collected and dialyzed against 0.05 mol/L N-cyclohexyl-3-aminopropanesulfonic acid (CAPS) at pH 10.3 (buffer B) for overnight. The sample was loaded onto a HiTrap Q-Sepharose Fast Flow (QSFF) column (GE Healthcare, Wuxi, China) that was pre-equilibrated with buffer B. Subsequently, the proteins were eluted with buffer B until OD_280_ < 0.05, followed by a gradient elution of NaCl (0–0.15 mol/L) at 1 mL/min. The purity of the fractions was verified by SDS-PAGE.

### SDS-PAGE, zymogram and molecular mass determination

SDS-PAGE was used to evaluate the purity and molecular mass of the protease. The protein bands were stained by Coomassie Brillaint Blue R-250. The molecular weight markers for electrophoresis included α-lactalbumin (14.4 kDa), trypsin inhibitor (20.1 kDa), carbonic anhydrase (29.0 kDa), ovalbumin (44.3 kDa), BSA (66.4 kDa) and phosphorylase B (97.2 kDa). Zymogram of the protease was analyzed using the SDS-polyacrylamide gel co-polymerized with 0.1% gelatin as described by Sun and co-workers [53]. The native molecular mass of the purified GsProS8 was estimated by a gel filtration chromatography via a Sephacryl S-100 column (1.0 × 100 cm). The purified protease and protein markers were eluted by 20 mM Tris-HCl buffer (at pH 8.0) containing 500 mM NaCl at a flow rate of 0.33 mL/min.

### Protease assay and protein determination

Protease activity was determined using casein as a substrate. Briefly, the protease solution (100 μL) was incubated with 2% (w/v) casein (100 μL) in 20 mM phosphate buffer (700 μL, pH 7.5) at 37 °C for 30 min. The reaction was terminated by trichloroacetic acid (10% w/v, 200 μL). After centrifugation at 10,000×*g* for 5 min, the supernatant (100 μL) was mixed with 600 μL alkaline reagent (0.4 mol/L Na_2_CO_3_: Folin-Ciocalteu reagent = 5:1 v/v) and incubated at 40 °C for 20 min. The absorbance was measured at 660 nm using a spectrophotometer (TU1901, Puxi General Instruments Co., Ltd., Beijing, China). The mixture without the protease was used as control. One unit of protease activity (U) was defined as the amount of the protease to liberate 1 μmol tyrosine per minute at the above conditions. Moreover, the protein concentration was measured by the Lowry method using BSA as standard. Specific activity was expressed as units per milligram of protein.

### Biochemical properties of GsProS8

The optimal pH for protease activity was evaluated at 40 °C in a pH range of 4.0–11.0. The following buffers (0.05 mol/L) were used: citrate (pH 4.0–5.0), acetate (pH 5.0–5.5), 2-(N-morpholino) ethanesulfonic acid (MES, pH 5.5–7.0), 4-(N-morpholino) propanesulfonic acid (MOPS, pH 7.0–8.5), and N-cyclohexyl-2-aminoethanesulfonic acid (CHES, pH 8.5–11.0). The pH stability was investigated after incubating the protease in various buffers (0.05 mol/L) at 40 °C for 30 min. The residual activity was determined by the standard assay.

The optimal temperature was determined in 0.05 mol/L of MOPS (pH 8.5) buffer at 30-70 °C. The thermostability was measured after incubating the protease at a temperature range of 30-80 °C for 30 min. The residual activity was determined by the standard assay.

The effect of several inhibitors on the protease activity was analyzed. The following inhibitors were used: pepstain A (at 0.01 mM and 0.02 mM), EDTA (at 1 mM and 4 mM), PMSF (at 1 mM and 4 mM), and iodoacetamide (at 1 mM and 4 mM). Moreover, the effect of metal ions on protease activity was examined in the presence of magnesium sulfate, calcium chloride, sodium chloride, nickel chloride, manganese sulfate, cobalt chloride, zinc sulfate, and copper sulfate at the final concentration of 0.001 mol/L.

Substrate specificity was evaluated at pH 8.5 and 50 °C using casein, whey protein, skim milk powder, hemoglobin, soybean protein isolate, BSA, protamine, myoglobin, azocasein, or gelatin (1% w/v). The protease activity was measured as described previously. The protease activity towards casein was determined to be 100%, to calculate the relative protease activities to other substrates.

### Kinetic parameters of GsProS8

The kinetic parameters of the purified GsProS8 were determined using Michaelis-Menten plot after measuring the protease activities at various concentrations of casein (4–16 mg/mL) and whey protein (8–25 mg/mL) at pH 8.5 and 50 °C for 5 min. The parameters included *K*_m_, *V*_max_, *k*_cat_ and *k*_cat_/*K*_m_, in which *K*_m_ and *V*_max_ were obtained from non-linear regression analysis by GraphPad Prism V.8 [54].

### Hydrolysis of whey protein by GsProS8

According to the preliminary test, pH, temperature, time, and whey protein concentration were identified as four critical factors to impact the hydrolysis of whey protein by GsProS8. Each factor was analyzed at three levels using an orthogonal experimental design (Table S2). In brief, whey protein (7–11%, w/v) was hydrolyzed by GsProS8 (200 U/mL) at designed temperature (40-60 °C) and pH (7.5–8.5) for 4–8 h in a temperature-control shaker (HZQ-X100, Suzhou, China) at 100 rpm, and terminated by heating at 85 °C for 10 min. The hydrolysate solutions were centrifuged (Refrigerated Centrifuge GL-20B, Shanghai, China) at 10,000×*g* for 10 min to obtain the supernatants for later analysis. Commercial proteases (200 U/mL, e.g., trypsin at pH 8.0 and 37 °C; Flavourzyme at pH 7.0 and 53 °C; Protamex at pH 7.5 and 40 °C; and Alcalase at pH 8.0 and 60 °C) were also applied in the hydrolysis of whey protein.

### Peptide content of whey protein hydrolysate

The peptide content in the whey protein hydrolysate was determined by o-phthalaldehyde (OPA) method as described by Church and co-workers [55] with slight modifications. Concisely, the supernatant of whey protein hydrolysate (25 μL) was added into OPA mixture (1 mL), which was prepared by 100 mM sodium tetraborate, 20% (w/w) SDS, and OPA solution (containing 0.02% w/v OPA, methanol, and β-mercaptoethanol). The incubation was then processed at room temperature for 8 min, and the absorbance of resulting solution at 340 nm (TU-1800PC spectrophotometer, Persee General Instrument Co. Ltd., Beijing, China) was recorded. Both distilled water and glutathione were performed as a blank and a standard, respectively. The peptide content was calculated based on the standard curve of glutathione.

### Antihypertensive activity of whey protein hydrolysate

Antihypertensive activity of whey protein hydrolysate was expressed as ACE inhibitory activity (%) and measured as described by Cushman and Cheung [56] with some modifications. Namely, the reaction of whey protein hydrolysate (2 mg/mL, 20 μL) with ACE (0.1 U/mL, 10 μL) in 120 μL of 0.1 M sodium borate buffer (containing 5 mM N-Hippuryl-His-Leu hydrate and 0.3 M NaCl at pH 8.3) was performed at 37 °C for 60 min. The reaction was then terminated by HCl (1 M, 150 μL) and ethyl acetate (1 mL), followed by centrifugation (GL-20B Refrigerated Centrifuge, Anting Scientific Instrument Co. Ltd., Shanghai, China) at 4000 rpm for 10 min at room temperature to collect the supernatant, which was dried at 105 °C for 30 min. The liberated hippuric acid was dissolved in the deionized water (0.5 mL) to record the absorbance at 228 nm (TU-1800PC, Persee General Instrument Co. Ltd., Beijing, China). The enzyme reaction without the whey protein hydrolysate and sodium borate buffer (0.1 M) containing substrate were used as a control and a blank, respectively. ACE inhibitory activity (%) was calculated as follows:

$$ \mathrm{ACE}\kern0.5em \mathrm{inhibitory}\kern0.5em \mathrm{activity}\left(\%\right)=\frac{{\mathrm{A}}_{\mathrm{b}}-{\mathrm{A}}_{\mathrm{a}}}{{\mathrm{A}}_{\mathrm{b}}-{\mathrm{A}}_{\mathrm{c}}}\times 100\% $$

where A_a_ is the absorbance of the sample supernatant, A_b_ is the absorbance of the control, and A_c_ is the absorbance of the blank. ACE inhibitory activity (%) was transformed to IC_50_ (mg/mL, half maximal inhibitory concentration) based on a plot of relative ACE inhibitory activity (%) against different dilutions (0.01–1.00%) of the hydrolyzed supernatant using non-linear regression analysis by GraphPad Prism V.8 (Fig. S5).

### Statistics

The experiments were performed in triplicate and expressed as the mean ± one standard deviation. A one-way analysis of variance (ANOVA) was carried out using Systat v10 software (San Jose, CA, USA) to analyze statistical differences on the protease activity, peptide content, and ACE inhibitory activity (IC_50_). *p* < 0.05 was considered significant.

## Supplementary Information

**Additional file 1.**

## Data Availability

The sequencing data of the alkaline serine protease gene (GsProS8) from *G. stearothermophilus* is presented on the NCBI database (GenBank accession number MW084977). The datasets used and/or analyzed during the current study are available from the corresponding author on reasonable request.

## Abbreviations

PMSF: Phenylmethylsulfonylfluoride
DFP: Diodopropyl fluorophosphate
GRAS: Generally recognized as safe
ACE: Angiotensin-I-converting enzyme
LB: Luria-Bertani
PCR: Polymerase chain reaction
ORF: Open reading frame
SDS-PAGE: Sodium dodecyl sulphate-polyacrylamide gel electrophoresis
SPFF: SP Fast Flow
CAPS: N-cyclohexyl-3-aminopropanesulfonic acid
QSFF: Q-Sepharose Fast Flow
BSA: bovine serum albumin
MES: 2-(N-morpholino) ethanesulfonic acid
MOPS: 4-(N-morpholino) propanesulfonic acid
CHES: N-cyclohexyl-2-aminoethanesulfonic acid
EDTA: Ethylenediaminetetraacetic acid
OPA: O-phthaladehyde
ANOVA: A one-way analysis of variance
