# Supplementary material for: High level expression and biochemical characterization of an alkaline serine protease from Geobacillus stearothermophilus to prepare antihypertensive whey protein hydrolysate
Source: BMC Biotechnol. 2021 Mar 11;21:21. doi: 10.1186/s12896-021-00678-7 (PMC7953746; doi:10.1186/s12896-021-00678-7)
Supplement: Supplementary file 1 — Additional file 1. [file 12896_2021_678_MOESM1_ESM.docx]

**High level expression and biochemical characterization of an alkaline serine protease from *Geobacillus stearothermophilus* to prepare antihypertensive whey protein hydrolysate**

**Chang Chang^1^, Siyi Gong^1^, Zhiping Liu^1^, Qiaojuan Yan^2^, Zhengqiang Jiang^1,^** *

^1^Key Laboratory of Food Bioengineering (China National Light Industry), College of Food Science and Nutritional Engineering, China Agricultural University, Beijing, China, 100083

^2^Beijing Advanced Innovation Center for Food Nutrition and Human Health, College of Engineering, China Agricultural University, Beijing, China, 100083

*Corresponding author:

**Zhengqiang Jiang**

College of Food Science and Nutritional Engineering, China Agricultural University

No. 17 Qinghua Donglu, Beijing, China, 100083

Tel: (+86) 010-62737689; Fax: (+86) 010-82388508

E-mail: [zhqjiang@cau.edu.cn](mailto:zhqjiang@cau.edu.cn)

**Supplementary figure captions**

Fig. S1 Polymerase chain reaction (PCR) amplification of GsProS8. Lane M, the marker 2000; lane 1, the PCR product (1149 bp)

Fig. S2 Extracellular protein analysis of GsProS8 by SDS-PAGE during fermentation. Lane M, the protein marker; lanes 1-9, the supernatants collected at 24 h, 48 h, 72 h, 96 h, 114 h, 128 h, 138 h, 144 h, and 146 h, respectively. The picture was captured using CBIO-GelPro system (Cbio, Beijing, China)

Fig. S3 Full-length gels of SDS-PAGE analysis for GsProS8 before and after purification. Lane M, the protein marker; lane 1, the crude proteins; lane 1a and 1b, the repeated analysis of crude proteins; lane 2, the proteins purified by ammonium sulphate precipitation; lane 2a, the repeated analysis of the proteins purified by ammonium sulphate precipitation; lane 3, the proteins purified by SPFF treatment; lane 4, the proteins purified by QSFF treatment; lane 4a and 4b, the QSFF purified proteins eluted by different gradients of NaCl (0-0.15 mol/L). The picture was captured using CBIO-GelPro system (Cbio, Bejing, China)

Fig. S4 Zymograms (a) and corresponding SDS-PAGE analysis (b) using different amounts of GsProS8 solutions. Lane M, the protein marker; lane 1, the zymogram prepared by 1 μL of protease solution; lane 2, the zymogram prepared by 5 μL of protease solution; lane 3, the zymogram prepared by 10 μL of protease solution; lane 4, the zymogram prepared by 20 μL of protease solution. The picture was captured using CBIO-GelPro system (Cbio, Beijing, China)

Fig. S5 IC_50_ plots to show angiotensin-I-converting enzyme (ACE) inhibitory activity of whey protein hydrolysates (WPH). WPT, whey protein hydrolysate prepared by trypsin at pH 8.0 and 37^o^C; WPF, whey protein hydrolysate prepared by Flavourzyme at pH 7.0 and 53^o^C; WPP, whey protein hydrolysate prepared by Protamex at pH 7.5 and 40^o^C; WPA, whey protein hydrolysate prepared by Alcalase at pH 8.0 and 60^o^C; WPG, whey protein hydrolysate prepared by GsProS8 at pH 8.0 and 60^o^C

**Table S1** Primers used for cloning and expression of the alkaline serine protease GsProS8

| **Primers** | **Nucleotide sequences (5’to 3’)** |
| --- | --- |
| GsProS8-F | CCGGAATTCATGAGAGGCAAAAAGGTATGGAT |
| GsProS8-R | AATGCGGCCGCTTACTGAGCTGCCGCCTGTA |
| GsProS8-IF | CAACTCAAGCTTTTGCCTCGAGCTCGGTACCCGGGATGAGAGGCAAAAAGGTATGGATCA |
| GsProS8-IR | CTGAAGCTAGCTTGCATGCCTGCAGTTACTGAGCTGCCGCCTGTACGTTGATCAGCCCTT |
| pWB980-VF | AAGGGCTGATCAACGTACAGGCGGCAGCTCAGTAACTGCAGGCATGCAAGCTAGCTTCAG |
| pWB980-VR | TGATCCATACCTTTTTGCCTCTCATCCCGGGTACCGAGCTCGAGGCAAAAGCTTGAGTTG |

**Table S2** Levels of key factors in the hydrolysis of whey protein using an orthogonal experimental design

| **Factor** | **Level** | | |
| --- | --- | --- | --- |
|  | **1** | **2** | **3** |
| pH | 7.5 | 8.0 | 8.5 |
| Temperature (^o^C) | 40 | 50 | 60 |
| Time (h) | 4 | 6 | 8 |
| Whey protein concentration (%) | 7 | 9 | 11 |

**
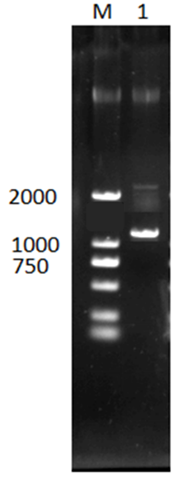
**

**Fig S1**

**
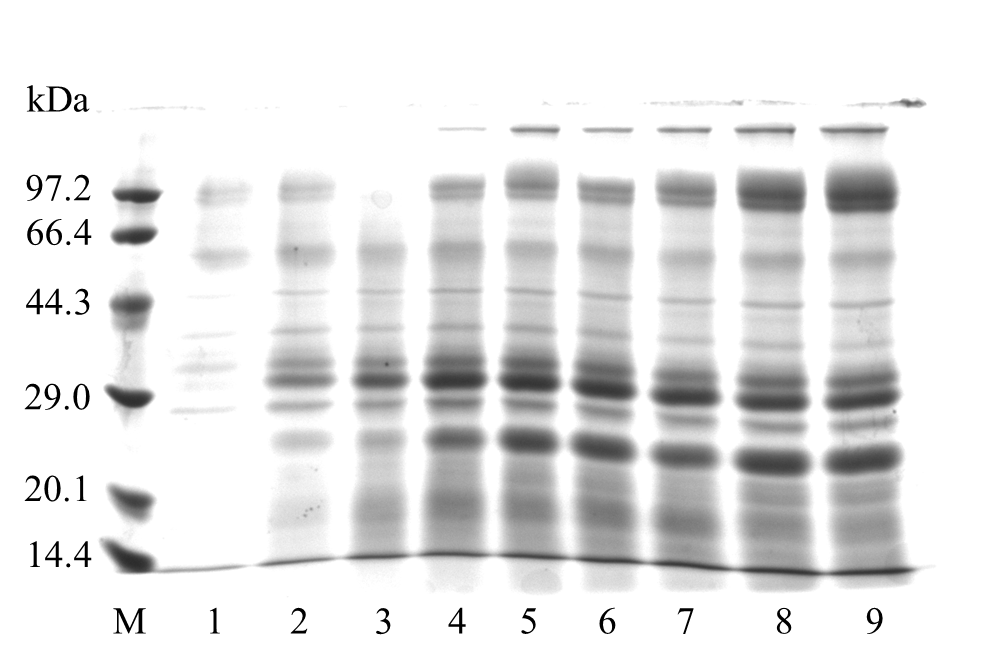
**

**Fig. S2**

**
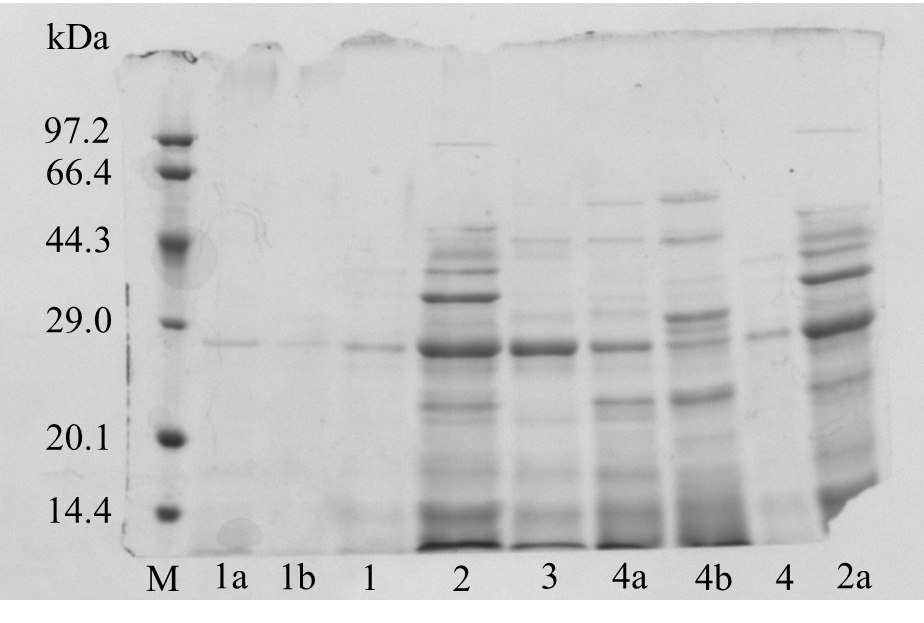
**

**Fig. S3**

**
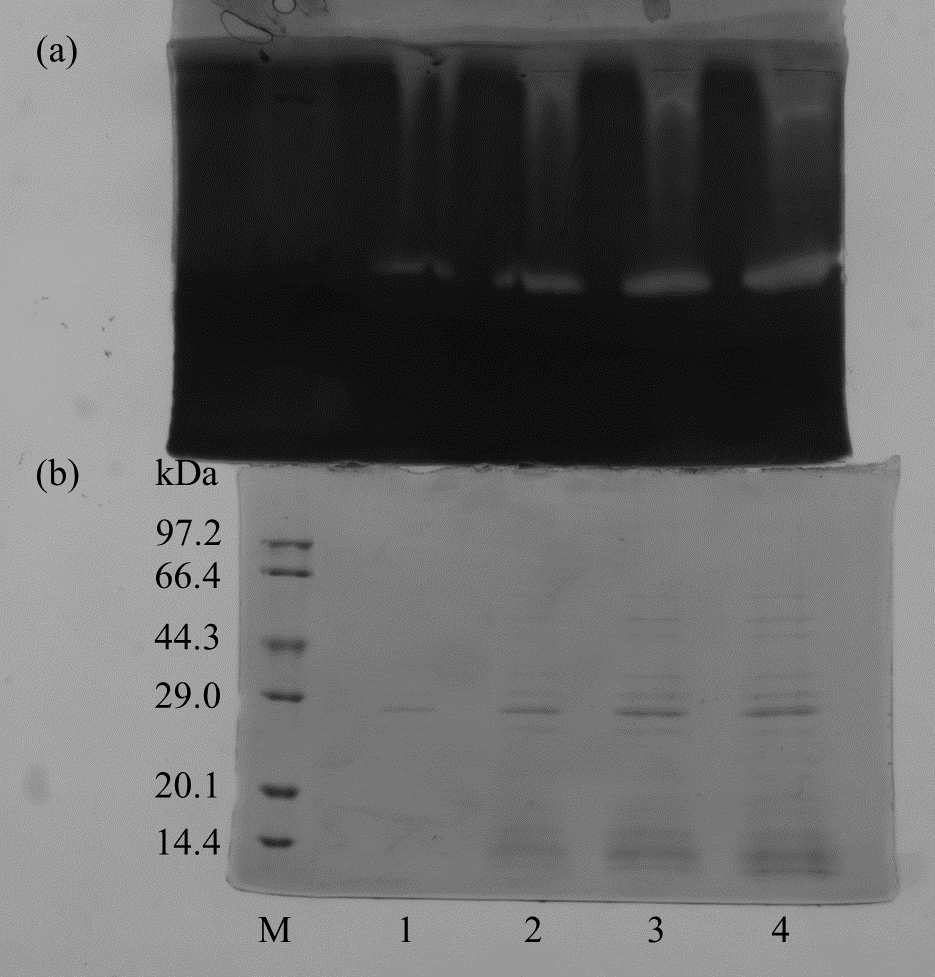
**

**Fig. S4**

**
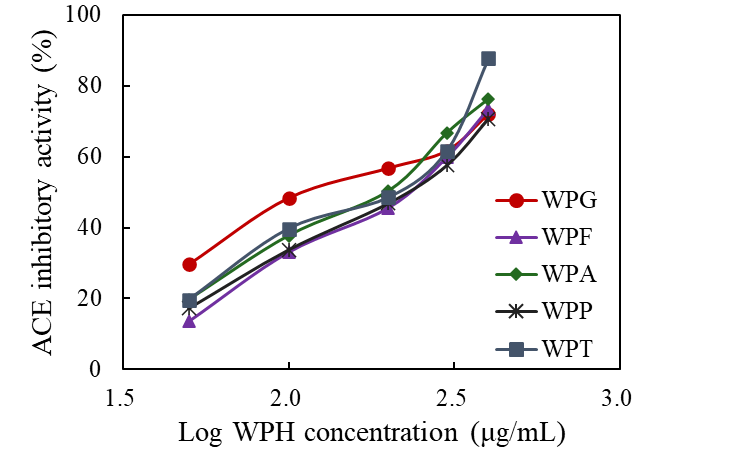
**

**Fig. S5**
